# Higher levels of total cholesterol/high-density lipoprotein cholesterol ratios are associated with an increased risk of suicidal behavior in children and adolescents with depressive disorders

**DOI:** 10.3389/fpsyt.2025.1557451

**Published:** 2025-03-12

**Authors:** Nana Sun, Zhiwei Liu, Liang Sun, Feng Sun, Longlong Sun, Jingjing Zhang, Rongchun Yang, Gaofeng Yao, Yun Liu

**Affiliations:** ^1^ Department of Psychiatry, Affiliated Psychological Hospital of Anhui Medical University, Hefei, China; ^2^ Department of Psychiatry, Hefei Fourth People’s Hospital, Hefei, China; ^3^ Department of Psychiatry, Anhui Mental Health Center, Hefei, China; ^4^ Department of Psychiatry, The Third People’s Hospital of Fuyang, Fuyang, China; ^5^ Department of Psychiatry, Fuyang Mental Health Center, Fuyang, China

**Keywords:** depressive disorder, suicidal ideation, suicide attempt, TG/HDL-C, TC/HDL-C, associated factors

## Abstract

**Objective:**

To explore the prevalence of suicidal ideation (SI) and suicidal attempt (SA) in inpatients with childhood and adolescent depressive disorders and the relationship between triglyceride/high-density lipoprotein cholesterol (TG/HDL-C), total cholesterol/high-density lipoprotein cholesterol (TC/HDL-C) ratios and SI, SA.

**Methods:**

A study was conducted involving 515 pediatric patients diagnosed with depressive disorders at the Third People’s Hospital of Fuyang. This research primarily focused on gathering basic demographic and clinical data. Through employing methods such as correlation analysis and logistic regression, the study aimed to identify factors linked to SI and SA among these individuals.

**Results:**

The prevalence of SI and SA was 20.0% (103/515) and 9.1% (47/515). Binary logistic regression highlighted several independent predictors for SI. A notable increase in the likelihood of SI was observed with an increase in the number of hospitalizations (Odds Ratio [*OR*]=1.65, *P*=0.025), a heightened TC/HDL-C ratio (*OR*=1.72, *P*=0.002), an escalated antidepressant dosage (*OR*=1.02, *P*=0.029), and elevated HAMD scores (*OR*=1.04, *P*=0.003). For SA, critical independent associated factors identified were an increased number of hospitalizations (*OR*=2.71, *P*<0.001), a higher TC/HDL-C ratio (*OR*=1.69, *P*=0.002), and greater HAMD scores (*OR*=1.06, *P*=0.003), particularly in children and adolescents diagnosed with depressive disorders.

**Conclusion:**

These findings suggest that monitoring lipid profiles, particularly the TC/HDL-C ratio, alongside careful management of antidepressant dosages and close observation of depressive symptoms, could be crucial in mitigating suicidal risks among this vulnerable population.

## 1 Introduction

Childhood and adolescence are critical periods of human growth and development, as well as essential stages of psychological development. Due to the immaturity of physiological and psychological development, emotions are often unstable and easily influenced by the external environment, which leads to adolescents having a high prevalence of depressive disorders. Recent global studies indicate a rising concern in adolescent mental health, with major depressive disorder exhibiting a one-year prevalence of 8% and a lifetime prevalence of 19% among teenagers (1). Additionally, data from a broader demographic encompassing children and adolescents aged 6 to 16 years old reveal a total mental illness prevalence of 17.5%, with depressive disorders specifically accounting for 3.0% of the cases (2). These statistics underscore the escalating incidence of depressive conditions in young populations, warranting urgent attention and comprehensive strategies to address this growing trend. Meanwhile, after the COVID-19 pandemic, depression, anxiety, and sleep disorders among children and adolescents were significantly elevated due to the effects of school closures, irregular life rhythms, and frequent use of daily social media (3). The Global Burden of Disease Study results show that depression disorder has the second highest age-standardized disability-adjusted life-years (DALYs) increase rate among the 25 major tertiary causes between 2010 and 2021 (4).

Approximately 10% of adolescents have attempted suicide, positioning it as the second leading cause of death in individuals aged 10-19 years (5). Depression further obviously elevated the likelihood of suicidal behavior (6). Surveys in Europe and the Americas have shown that the total cost and burden of suicide and attempted suicide is enormous, with serious adverse effects on families and socio-economics (7, 8). Historical evidence indicated that suicidal ideation is prevalent in adolescents with depression (9). In former study, Liu et al. observed a noteworthy prevalence of suicidal ideation and attempts among adolescents diagnosed with depressive disorders, with rates of 17.2% and 9.9% respectively (10). Meanwhile, adolescence depression is a significant risk factor for suicide, and more than thirty percent of adolescent deaths in the United States are caused by suicide (11). Depression and high suicide rates among adolescents lead to pressure on health resources, increased risk of family breakdown, increased burden on the education system and loss of productivity. Long-term impacts on socio-economic development exacerbate the intergenerational transmission of psychological problems, and early intervention needs to be strengthened in order to reduce the social costs. This finding underscores the critical intersection of depression and suicidal tendencies within this demographic.

Previous literature pointed out that dyslipidemia and metabolism-related problems are more common in the depressed population, which becomes a vital obstacle affecting clinical treatment and patients’ recovery prognosis. A prior investigation revealed that among Chinese adolescents, the prevalence rate of dyslipidemia stands at 25.3%. This condition manifests most frequently as hypercholesterolemia at 4.1%, hypertriglyceridemia at 8.5%, elevated low-density lipoprotein cholesterol (LDL-C) at 5.3%, and reduced high-density lipoprotein cholesterol (HDL-C) at 6.8% (12). Further research indicates a higher occurrence of dyslipidemia among individuals with mental challenges (13), with abnormal lipid profiles closely linked to more severe clinical manifestations (14). Another study showed that high levels of total cholesterol (TC) and triglyceride (TG) in adolescents with depressive disorder tended to associated with suicidal ideation and suicide attempts, while low HDL-C levels were significantly related to suicide attempts (15). Disorders of lipid metabolism and their association with patients’ symptom relief and clinical recovery are of increasing interest.

Specifically, studies have highlighted a significant correlation between lipid profiles and suicidal behavior in depressed individuals. Individuals at high risk for suicide tend to exhibit signs of dyslipidemia (16). Further studies have shown that individuals who have recently attempted suicide typically exhibit lower TG levels and higher levels of HDL-C compared to those who have not attempted suicide (17). In addition, among major depressive disorder (MDD) outpatients with suicide attempts, there was a trend toward higher levels of TC and LDL-C, as well as significantly lower levels of HDL-C, compared with those who did not attempt suicide (18). These findings suggest a complex relationship between lipid dysregulation and suicidal ideation in depressed individuals, pointing to the potential of lipid profiles as biomarkers for clinical suicide risk assessment. In addition to this, associations between TG/HDL-C ratio and dyslipidemia levels have been noted and correlated with clinical psychiatric symptoms (19, 20), but these potential associations between suicidal behaviors and lipid profiles, especially the TG/HDL-C and TC/HDL-C ratios, are still under-explored. In summary, although extensive research has addressed depression in children and adolescents, knowledge of suicidal ideation and attempts in this population remains limited. The aim of the present study was to provide insight into the prevalence of suicidal ideation and attempts among children and adolescents with depression, as well as the potential link between these suicidal behaviors and lipid profiles. By delving into these key aspects, this study aims to shed light on the mental health challenges of this vulnerable group and the biochemical parameters that may affect them, providing a scientific basis for future clinical interventions.

## 2 Objectives and methods

### 2.1 Subjects

This research focused on a specific group of pediatric patients diagnosed with depressive disorders, who were admitted to the Third People’s Hospital of Fuyang over the year spanning January to December 2021. The selection of participants was strictly defined by the following inclusion criteria: individuals aged between 8 and 18 years who conformed to the depressive disorder diagnosis as outlined in the fifth edition of the Diagnostic and Statistical Manual of Mental Disorders (DSM-V). Exclusion criteria were meticulously set to isolate the effects of depressive disorders from other confounding variables. (1) Patients with concurrent mental health disorders, such as anxiety or conduct disorders. (2) Additionally, those suffering from severe neurological or physical conditions were also not included in this research. Prior to initiation of the study, both the participants and their guardians provided their consent, having been fully informed about the study’s aims and procedures. All procedures contributing to this work comply with the ethical standards of the relevant national and institutional committees on human experimentation and with the Helsinki Declaration of 1975, as revised in 2013. All procedures involving human subjects/patients were approved by the Medical Ethics Committee of the Third People’s Hospital of Fuyang [approval number: 2018-340-10].

### 2.2 General information and biochemical indicators

#### 2.2.1 General information and clinical data collection

A unified questionnaire was designed to record the sociodemographic data, such as sex, age, and other information: onset age, the category, and dosage of the medication taken by the patients, and blood glucose and lipid indicators were recorded. The therapeutic dose of antidepressants taken by the patients was converted to fluoxetine equivalents (21).

#### 2.2.2 Biochemical indicators

Laboratory technicians assessed fasting blood glucose (FBG), total cholesterol (TC), triglycerides (TG), high-density lipoprotein cholesterol (HDL-C), and low-density lipoprotein cholesterol (LDL-C) levels in all subjects. Subsequently, researchers computed the ratios of TG/HDL-C and TC/HDL-C.

#### 2.2.3 Hamilton depression scale

The researcher used 24-item HAMD to assess the depressive symptoms of the enrolled subjects. The interclass correlation coefficient (ICC) was 0.92 after rigorously training the rater. In addition, the HAMD, as an other-rated scale, can be explained by asking the investigator directly to ensure the accuracy of the survey results due to the age of the included subjects, if there are any problems that are difficult to understand.

#### 2.2.4 Suicidal ideation, suicidal attempt

We set three subjective questions to assess whether patients had SI, or SA during the past month. (1) “Have you seriously considered suicide or killing yourself?”; (2) “Have you made a detailed plan to commit suicide?”; “Are you trying to kill yourself? And some specific measures were taken (wrist cutting/medication/suicide by jumping/drowning, etc.)” The answer to each question was “yes” or “no”. If the patient answers “yes” to only one or both of the first two questions, suicidal ideation is considered to be present. If the patient answered yes to the third question, with or without the first two questions, a suicide attempt was identified.

### 2.3 Statistical methods

The analysis of the data was performed using the SPSS 23.0 software package, sourced from Chicago, Illinois, United States. We expressed the prevalence of suicidal ideation and attempts as a percentage. To begin, we evaluated differences in sociodemographic, clinical, and biomarker variables across groups. This was achieved using independent t-tests for continuous variables and Chi-square tests for categorical variables. Further, we employed Analysis of Covariance (ANCOVA) to assess differences in TG/HDL-C and TC/HDL-C ratios between groups with and without suicidal ideation or attempts. This analysis adjusted for demographic and clinical variables that showed significant differences between the groups. Additionally, the Spearman rank correlation method facilitated the examination of relationships between suicidal ideation (SI), suicidal attempts (SA), and variables included in the study. Moreover, socio-demographic variables that demonstrated a P-value of less than 0.05 in the univariate logistic regression analysis were selected for the multivariable logistic regression analysis. This step was crucial to ascertain whether the TG/HDL-C and TC/HDL-C ratios stood as independent related factors for suicidal ideation or attempts among children and adolescents diagnosed with depressive disorders. We considered statistical differences significant at a P-value less than 0.05.

## 3 Results

### 3.1 Intergroup comparison of general information and biological indicators

The prevalence of children with depressive disorders with SI and SA was 20.0% (103/515) and 9.1% (47/515). The study revealed notable disparities across various parameters including total duration, frequency of admissions, TC, HDL-C, LDL-C, the TC/HDL-C ratio, antidepressant dosage, and HAMD scores when comparing groups with suicidal ideation (SI) to those without (*P*<0.05). There was a statistical difference between total duration, number of admissions, LDL-C, TG/HDL-C ratio, TC/HDL-C ratio, antidepressant dosage, and HAMD scores between groups in the accompanied SA group and those without SA (*P*<0.05). The ANCOVA analysis confirmed that the disparity in TC/HDL-C ratios (*F* = 7.14, *P* = 0.008) between the groups with and without SI was statistically significant, even after adjusting for demographic and clinical variables that varied notably between the groups. Likewise, ANCOVA showed that differences in TC/HDL-C (*F* = 6.12, *P* = 0.014), and TG/HDL-C (*F* = 8.32, *P* = 0.004) between the two groups with or without SA remained significant. As shown in **Table 1**.

**Table 1:** Comparison of general information and biological indexes between the group without SI and the group with SI, and the group without SA and the group with SA.

Variables	Without SI (*n*=412)	SI (*n*=103)	χ^2^ /F	*P*	Without SA (*n*=468)	SA (*n*=47)	χ^2^ /F	*P*
Age (years)	15 (13, 16)	15 (14, 17)	-1.357	0.175^b^	15 (13, 16)	15 (14, 17)	-0.510	0.610 ^b^
Gender								
Male	122 (29.6)	24 (23.3)	1.616	0.204^c^	138 (29.5)	8 (17.0)	3.268	0.071^c^
Female	290 (70.4)	79 (76.7)			330 (70.5)	39 (83.0)		
Onset age (years)	14 (12, 15)	14 (12, 15)	-0.027	0.979 ^b^	14 (12, 15)	13.3 ± 1.7	-1.365	0.172 ^b^
Age of first admission (years)	15 (13, 16)	15 (14, 16)	-1.041	0.298 ^b^	15 (13, 16)	14.9 ± 1.6	-0.088	0.930 ^b^
Total duration (months)	12 (4, 24)	12 (10, 24)	-3.425	0.001 ^b^	12 (5, 24)	12 (12, 24)	-3.458	0.001 ^b^
Number of admissions	1 (1, 1)	1 (1, 1)	-3.891	<0.001^b^	1 (1, 1)	1 (1, 2)	-6.134	<0.001^b^
BMI	20.3 (18.3, 23.2)	20.7 (18.0, 24.2)	-0.126	0.900 ^b^	20.2 (18.2, 23.4)	21.0 (18.2, 25.3)	-1.424	0.155 ^b^
Haematological index								
FBG (mmol/L)	5.04 ± 0.48	4.97 ± 0.59	1.396	0.163^a^	5.03 ± 0.47	5.06 ± 0.76	-0.433	0.665 ^a^
TG (mmol/L)	0.84 (0.65, 1.17)	0.93 (0.65, 1.40)	-1.524	0.127 ^b^	0.84 (0.65, 1.18)	1.28 ± 0.88	-1.922	0.055 ^b^
TC (mmol/L)	3.68 (3.25, 4.20)	3.97 ± 0.76	-2.726	0.006 ^b^	3.75 ± 0.82	4.00 ± 0.82	-1.967	0.050 ^a^
HDL-C (mmol/L)	1.47 (1.31, 1.69)	1.45 ± 0.36	-2.187	0.029 ^b^	1.46 (1.30, 1.69)	1.43 ± 0.33	-1.630	0.103 ^b^
LDL-C (mmol/L)	2.23 (1.91, 2.59)	2.46 ± 0.56	-3.087	0.002 ^b^	2.25 (1.92, 2.61)	2.50 ± 0.59	-2.218	0.027 ^b^
TG/HDL-C	0.57 (0.41, 0.83)	0.66 (0.41, 1.05)	-1.910	0.056 ^b^	0.57 (0.41, 0.83)	0.99 ± 0.82	-2.252	0.024 ^b^
TC/HDL-C	2.46 (2.13, 2.83)	2.83 ± 0.70	-4.034	<0.001^b^	2.50 (2.15, 2.87)	2.91 ± 0.77	-3.034	0.002^b^
Antidepressants medication (%)								
None	17 (4.1)	6 (5.8)	0.559	0.756^c^	19 (4.1)	4 (8.5)	2.697	0.260^c^
Monotherapy	375 (91.0)	92 (89.3)			425 (90.8)	42 (89.4)		
Polypharmacy	20 (4.9)	5 (4.9)			24 (5.1)	1 (2.1)		
Antidepressant dosage (mg/d)	20.3 (20.0, 40.0)	29.2 (20.3, 40.6)	-3.050	0.002 ^b^	20.3 (20.0, 40.0)	40.0 (20.3, 40.6)	-2.269	0.023 ^b^
HAMD	20.0 (17.3, 30.0)	26.7 ± 8.1	-3.964	<0.001^b^	20.0 (18.0, 30.0)	28.3 ± 7.9	-3.834	<0.001^b^

BMI, body mass index; FBG, fasting blood glucose; TG, triglyceride; TC, total cholesterol; HDL-C, high-density lipoprotein cholesterol; LDL-C, low-density lipoprotein cholesterol; HAMD, Hamilton Depression Scale. ^a^ Independent samples t-test; ^b^ Mann-Whitney U-test; ^c^ Pearson chi-square.

### 3.2 Correlation analysis of SI and SA behaviors with general information and biochemical indicators

The study revealed a significant positive correlation of SI behavior with TC, LDL-C, the ratio of TC/HDL-C, and HAMD scores. Conversely, a negative correlation was observed with HDL-C levels. Similarly, SA behavior was positively correlated with LDL-C, the ratio of TG/HDL-C, TC/HDL-C, and HAMD scores. These findings are detailed in **Table 2**.

**Table 2:** Correlation analysis of SI, SA with general information and biochemical indicators.

Variables	SI		SA	
	r	P	r	P
Age (years)	0.060	0.175	0.022	0.610
Gender	0.056	0.204	0.080	0.071
Onset age (years)	0.001	0.979	-0.060	0.172
Age of first admission (years)	0.046	0.298	0.004	0.930
Total duration (months)	0.151	0.001	0.153	0.001
Number of admissions	0.172	<0.001	0.271	<0.001
BMI	0.006	0.900	0.063	0.155
FBG	-0.084	0.056	-0.011	0.796
TG (mmol/L)	0.067	0.128	0.085	0.055
TC (mmol/L)	0.120	0.006	0.073	0.098
HDL-C (mmol/L)	-0.096	0.029	-0.072	0.103
LDL-C(mmol/L)	0.136	0.002	0.098	0.026
TG/HDL-C	0.084	0.056	0.099	0.024
TC/HDL-C	0.178	<0.001	0.134	0.002
Antidepressants medication	-0.022	0.615	-0.070	0.111
Antidepressant dosage (mg/d)	0.138	0.002	0.103	0.023
HAMD	0.175	<0.001	0.169	<0.001

BMI, body mass index; FBG, fasting blood glucose; TG, triglyceride; TC, total cholesterol; HDL-C, high-density lipoprotein cholesterol; LDL-C, low-density lipoprotein cholesterol; HAMD, Hamilton Depression Scale.

### 3.3 Independent influences of depression disorder patients with SI and SA behaviors

Prior to conducting the logistic regression model analysis, the presence of multicollinearity between the study included independent variables was tested. The analysis revealed that all variables had tolerance levels exceeding 0.1 and variance inflation factors (VIF) below 10, indicating no significant multicollinearity concerns between the variables that differed between groups. Using SI behavior (without SI=0, with SI=1) as the dependent variable, regression analysis revealed several independent related factors associated with SI among patients. Notably, an increased number of hospital admissions presented a significant related (*OR*=1.65, *95% CI:* 1.06-2.56, *P*=0.025). Additionally, the TC/HDL-C ratio was identified as another crucial factor (*OR*=1.72, *95% CI:* 1.22-2.42, *P*=0.002). The dosage of antidepressants also correlated with SI, showing an OR of 1.02 (*95% CI*: 1.00-1.04, *P*=0.029), alongside the HAMD scores, which had an OR of 1.04 (95% CI: 1.01-1.07, *P*=0.003). Further details are provided in **Table 3**.

**Table 3:** Dichotomous logistic analysis of childhood and adolescent depressive disorder patients with SI.

Variables	B	Wald	P	OR	*95.0% C.I.*	
					lower bound	Upper bound
Number of admissions	0.502	5.006	0.025	1.65	1.06	2.56
TC/HDL-C	0.540	9.549	0.002	1.72	1.22	2.42
Antidepressant dosage	0.019	4.776	0.029	1.02	1.00	1.04
HAMD	0.041	8.882	0.003	1.04	1.01	1.07
Constant	-5.005	54.723	0.000	.007		

TC, total cholesterol; HDL-C, high-density lipoprotein cholesterol; HAMD, Hamilton Depression Scale.

In a similar vein, exploring SA behavior among children and adolescents with depressive disorders, the research utilized SA behavior as the dependent variable (coded as without SA=0, with SA=1). The findings highlighted that a higher number of hospitalizations significantly associated with SA, with an OR of 2.71 (*95% CI*: 1.62-4.54, *P*<0.001). The TC/HDL-C ratio and HAMD scores were also significant, recording ORs of 1.69 (*95% CI*: 1.06-2.70, *P*=0.028) and 1.06 (*95% CI*: 1.02-1.11, *P*=0.003) respectively. These results are detailed in **Table 4**.

**Table 4:** Dichotomous logistic analysis of childhood and adolescent depressive disorder patients with SA.

Variables	B	Wald	P	OR	*95.0% C.I.*	
					lower bound	Upper bound
Number of admissions	0.997	14.430	<0.001	2.71	1.62	4.54
TC/HDL-C	0.524	4.820	0.028	1.69	1.06	2.70
Antidepressant dosage	0.016	1.851	0.174	1.02	0.99	1.04
HAMD	0.060	8.949	0.003	1.06	1.02	1.11
Constant	-6.570	49.834	.000	.001		

TC, total cholesterol; HDL-C, high-density lipoprotein cholesterol; HAMD, Hamilton Depression Scale.

## 4 Discussion

The present study indicated that the prevalence of children with depressive disorders with SI and SA was 20.0% (103/515) and 9.1% (47/515). A study involving five Southeast Asian countries found that the overall rate of SI and SA among adolescents was 11.7% and 2.4% (22), while the incidence of SI among Chinese teenagers was 15.7% (23). Previous studies noted that the incidence of SA was as high as 20.1% in MDD outpatients (18). There are numerous factors influencing suicidal behavior in the child and adolescent population. From a psychological point of view, insecure attachment patterns, unhealthy psychological status of the mother, and psychological problems of the children themselves are closely related to the high risk of suicide (24, 25). In addition, from a behavioral perspective, the use of drugs or hazardous substances by the patient and living in poverty since childhood may increase suicidal behavior in children and adolescents (25, 26). Furthermore, molecular results show that abnormal genetic structure and abnormal levels of biochemical indicators can also adversely affect suicidal behavior in this population (24, 27). In addition, as the main goal of this study, lipid levels are significantly associated with depression or depressive symptoms (28), and impaired cholesterol metabolism may alter neuronal homeostasis *in vivo*, subsequently affecting the onset and progression of neuropsychiatric disorders (29). Furthermore, there may be a substantial correlation between high TG levels and a high risk of depressive symptoms (30). Lipid profiles, particularly reduced total cholesterol levels, have been identified as significant predictors of suicidal tendencies among individuals suffering from depression. This correlation suggests a heightened suicide risk in patients with lower cholesterol levels (16).

Recent studies have illuminated intriguing biochemical correlates associated with suicidal behavior, particularly focusing on lipid profiles. Specifically, research has consistently shown that levels of TC, TG, and LDL-C are generally lower in individuals who have SA compared to healthy controls. Conversely, high-density lipoprotein cholesterol (HDL-C) levels tend to be elevated in this demographic (31). A noteworthy aspect of these findings is the differential impact of HDL-C on suicidal risk across genders. For instance, among females, lower levels of HDL-C have been linked to an increased incidence of SA (32). Further longitudinal analyses reinforce this correlation, revealing that women with diminished HDL-C levels face a heightened risk of subsequent suicide attempts within a year (31). This pattern suggests that HDL-C could potentially serve as a biomarker for forecasting suicide risk in the future. The relationship between lipid levels and mental health extends beyond suicidal tendencies to encompass depressive disorders. Individuals exhibiting below-median levels of HDL-C are at a 2.4-fold greater risk of developing depressive disorders compared to those with higher HDL-C concentrations (33). This association is further complicated when considering first-episode depression, where patients demonstrate significantly elevated TC, LDL-C, and TG levels if they also display suicidal behaviors (34). Moreover, the interplay between lipid profiles and suicidal actions appears to vary over time. For instance, in patients with MDD who have recently attempted suicide, a pattern emerges showing lower TG and higher HDL-C levels compared to those with no recent suicide attempts (17). Additionally, in first-episode MDD patients, there is a positive correlation between TC levels and suicidal behavior, whereas HDL-C levels are inversely related (35). These findings collectively underscore the complex and significant role that lipid metabolism may play in both the pathophysiology of depressive and suicidal conditions. The potential of using lipid profiles, particularly HDL-C, as predictive markers for psychiatric interventions offers a promising avenue for future research and clinical application, aiming to mitigate the risks associated with these severe mental health challenges.

In recent researches, scientists explored the lipid profiles in patients with depressive disorder who exhibit SI and SA. The findings indicate that certain lipid fractions are intricately linked with both the presence and severity of depressive symptoms and suicidal behaviors. Previous analysis revealed that patients with prior suicidal ideation demonstrated significantly lower triglyceride (TG) levels (36). Furthermore, suicidal tendencies in MDD patients correlate strongly with diminished levels of TC and LDL-C (37). Contrarily, elevated levels of TG, TC, and LDL-C are associated with more intense depressive manifestations (38). Additionally, a substantial relationship was observed between TC, HDL-C, LDL-C, and the frequency of suicidal attempts among these individuals (39). Specifically, the study identified a positive correlation between suicidal ideation and higher ratios of TC to HDL-C, LDL-C, and a negative correlation with HDL-C levels alone. Similarly, suicidal attempts correlated positively with increased levels of LDL-C and the ratios of TG to HDL-C, and TC to HDL-C. These correlations underscore a clearer linkage between lipid profiles, depressive states, and suicidal behaviors in MDD patients. The current dataset substantiates the hypothesis that elevated levels of TG, TC, and LDL-C, alongside reduced HDL-C levels, may elevate the risk of suicidal behaviors. This insight enhances our understanding of the biochemical factors that may influence the psychiatric and behavioral outcomes in depressive disorders.

It has been noted that insulin resistance and disorders of glucolipid metabolism may be more common in depressed patients with suicidal behavior (34). TG/HDL-C and TC/HDL-C ratios are increasingly recognized for their utility in predicting insulin resistance, serving pivotal roles in clinical assessments (40). Research highlights that male students exhibiting insulin resistance typically present with elevated TC/HDL-C ratios compared to their counterparts without insulin resistance, establishing a positive correlation between the ratio and insulin sensitivity (41). Similarly, in females, TG/HDL-C levels have shown a direct relationship with insulin resistance (41). Furthermore, individuals suffering from affective disorders demonstrate higher TG/HDL-C levels than the general healthy population (42). Notably, in cases of manic episodes, a significant positive correlation exists between heightened TG/HDL-C levels and increased mania scores (20), underscoring the potential of these lipid ratios in reflecting broader metabolic and psychological disturbances. Secondly, previous research has found that as TG/HDL-C levels rise, the body’s LDL-C levels increase, leading to a higher risk of atherosclerosis in the body (19). In addition, follow-up investigations showed that patients with depressive disorders who were first unmedicated had increased TG levels and atherosclerotic indices after 12 weeks of antidepressant treatment, indicating a link between the link of clinical depressive symptoms and changes in adverse lipid indices (43). Our research indicates a notable correlation between elevated TC/HDL ratios and SI. Furthermore, increased hospital admissions, heightened TC/HDL ratios, and elevated HAMD scores independently contribute to the risk of SA, aligning with prior scholarly findings. In sum, the intricate lipid metabolism in patients with depressive disorders is more than just abnormal levels of a single indicator. Notably, the alterations observed in the TG/HDL-C and TC/HDL-C ratios and their profound association with depressive symptoms and suicidal tendencies underscore the complex interplay between lipid profiles and mental health dynamics.

The present study found a positive correlation between more hospitalizations and SI and SA, in addition to a strong relationship between lipid levels and suicidal behavior. Patients with depressive disorders who were hospitalized multiple times were found to have significantly more severe depressive symptoms and a higher risk of suicide (44). Also, depressed patients with more hospitalizations responded less well to antidepressants and had more prominent residual symptoms (e.g., fatigue, lack of pleasure), which may be related to neurobiological alterations (45). For patients with refractory depression, the number of hospitalizations was positively associated with symptom severity, co-morbid anxiety disorders, and overall functional impairment, suggesting that more frequent hospitalizations may reflect the degree of disease chronicity (46). Thus, more frequent hospitalizations may imply more severe clinical symptoms, poorer treatment outcomes, and, as previously known, more severe depressive symptoms are associated with lipid metabolism disorders that are more common in patients (28). In summary, the number of hospitalizations and abnormal lipid metabolism may combine to influence self-injurious behavior in patients with depressive disorders.

This research indicates a heightened occurrence of suicidal thoughts and behaviors among adolescents suffering from depressive disorders. It underscores the necessity for clinicians to rigorously assess and address suicide risk during diagnostic and therapeutic processes, in order to reduce the incidence of serious self-injury, suicide and other adverse events. A robust correlation exists between dyslipidemia in patients and their experiences of suicidal ideation or attempts. This connection underscores the necessity of closely monitoring dyslipidemia, alongside related conditions such as abnormal glucose metabolism and obesity, when addressing psychiatric symptoms in these individuals. In managing depressive disorders among adolescents, it is critical to tailor treatments that minimally impact glucose and lipid metabolism. This approach not only mitigates metabolism-related adverse effects but also enhances patient compliance and overall quality of life throughout the recovery process.

This research presents several limitations: (1) The design as a cross-sectional survey inhibits our ability to infer causality or establish a temporal sequence between SI, SA, and variations in lipid concentrations or ratios such as TG/HDL-C and TC/HDL-C. This ambiguity leaves open the question of whether changes in lipid profiles precede suicidal behaviors or if these behaviors influence lipid abnormalities. The causality needs to be further explored in retrospective or follow-up cohort studies, such as setting up multi-nodal follow-up with baseline, three-month, six-month, one-year, and two-year time-points, with repeated data collection for causality analysis; (2) The variables included in this study, such as BMI, blood lipid level and medication type, may also be confounding factors affecting patients’ suicidal behavior. Although no statistical significance was found in the results analysis, their independent effects on suicidal behavior and their interactions are still worthy of further exploration; (3) We fully recognize the important regulatory role of lifestyle factors such as dietary patterns and exercise status on lipid metabolism, but as the study mainly focused on the core correlations between biomarkers and mental health outcomes, in order to avoid the interference of multifaceted confounding factors in the preliminary exploration of the mechanisms, we prioritized to ensure the precision of measurements and depth of analysis of the core variables in the face of the limited completeness of the available data and the limited research resources; (4) The number of participants in the study needs to be further expanded to improve the generalizability and clinical significance of the results. And, the use of antipsychotics or antidepressants may lead to abnormalities in blood lipid levels or body weight in patients, but patients who were not on medication for the first time were not specifically selected for inclusion in this study. In the future, we may consider including these patients in studies to further control for confounding variables.

## 5 Conclusion

Suicidal ideation and behavior now represent a critical worldwide public health challenge. This study examines the prevalence of suicidal ideation and attempts in hospitalized children and adolescents with depressive disorders, providing a partial reference to the currently scarce epidemiologic data; integrating clinical psychiatric symptoms with biochemical index testing to provide quantifiable physiological reference values; and research findings to aid clinical intervention and provide potentially useful biological early warning indicators for suicide risk identification. Our investigation revealed a pronounced prevalence of SI and SA among adolescents suffering from depressive disorders, closely linked to dysregulated lipid profiles, particularly the ratios of TG/HDL-C and TC/HDL-C. Through regression analysis, SI was observed with an increase in the number of hospitalizations, a heightened TC/HDL-C ratio, an escalated antidepressant dosage, and elevated HAMD scores. For SA, critical independent related factors identified were an increased number of hospitalizations, a higher TC/HDL-C ratio, and greater HAMD scores, particularly in children and adolescents diagnosed with depressive disorders. Thus, elevated TC/HDL-C emerged as a novel and independent predictor of both SI and SA, underscoring its potential as a biomarker for suicide behavior in this vulnerable demographic.

## Data Availability

The raw data supporting the conclusions of this article will be made available by the authors, without undue reservation.
